# Visual Field Prognosis From Macula and Circumpapillary Spectral Domain Optical Coherence Tomography

**DOI:** 10.1167/tvst.13.6.10

**Published:** 2024-06-17

**Authors:** Davide Scandella, Mathias Gallardo, Serife S. Kucur, Raphael Sznitman, Jan Darius Unterlauft

**Affiliations:** 1 ARTORG Center, Universität Bern, Bern, Switzerland; 2 PeriVision SA, Epalinges, Switzerland; 3 Department of Ophthalmology, Inselspital Universitätsspital Bern, Bern, Switzerland

**Keywords:** glaucoma, visual field, deep learning

## Abstract

**Purpose:**

To explore the structural-functional loss relationship from optic-nerve-head– and macula-centred spectral-domain (SD) Optical Coherence Tomography (OCT) images in the full spectrum of glaucoma patients using deep-learning methods.

**Methods:**

A cohort comprising 5238 unique eyes classified as suspects or diagnosed with glaucoma was considered. All patients underwent ophthalmologic examination consisting of standard automated perimetry (SAP), macular OCT, and peri-papillary OCT on the same day. Deep learning models were trained to estimate G-pattern visual field (VF) mean deviation (MD) and cluster MD using retinal thickness maps from seven layers: retinal nerve fiber layer (RNFL), ganglion cell layer and inner plexiform layer (GCL + IPL), inner nuclear layer and outer plexiform layer (INL + OPL), outer nuclear layer (ONL), photoreceptors and retinal pigmented epithelium (PR + RPE), choriocapillaris and choroidal stroma (CC + CS), total retinal thickness (RT).

**Results:**

The best performance on MD prediction is achieved by RNFL, GCL + IPL and RT layers, with *R*^2^ scores of 0.37, 0.33, and 0.31, respectively. Combining macular and peri-papillary scans outperforms single modality prediction, achieving an *R*^2^ value of 0.48. Cluster MD predictions show promising results, notably in central clusters, reaching an *R*^2^ of 0.56.

**Conclusions:**

The combination of multiple modalities, such as optic-nerve-head circular B-scans and retinal thickness maps from macular SD-OCT images, improves the performance of MD and cluster MD prediction. Our proposed model demonstrates the highest level of accuracy in predicting MD in the early-to-mid stages of glaucoma.

**Translational Relevance:**

Objective measures recorded with SD-OCT can optimize the number of visual field tests and improve individualized glaucoma care by adjusting VF testing frequency based on deep-learning estimates of functional damage.

## Introduction

Glaucoma is among the leading causes of irreversible blindness worldwide, with an estimated prevalence of 3.5% in the population aged 40 to 80 years and an estimated number of over 110 million people affected by 2040.^1^^,^^2^ In glaucoma, the progressive demise of retinal ganglion cells (RGC) leads to alterations of the retinal layers containing RGC soma, axons, and dendrites, which can be measured in the central retina or around the optic nerve head using optical coherence tomography (OCT). Functionally, loss of RGC leads to visual field defects (i.e., scotomas), which follow distinct development stages. These can be measured and monitored using visual field testing. Perimetry has several disadvantages, however. First, it is highly subjective and depends strongly on the patient's concentration and alertness, generating highly variable results. Second, it was estimated that finding visual field defects using perimetry in glaucoma is only possible when >50% of ganglion cells are already missing.^3^

Although OCT was initially used to diagnose predominantly macular pathologies (age-related macular degeneration, epiretinal gliosis, etc.), technological developments have yielded spatial resolutions of 7 µm imaging coupled with automatic retinal layer segmentation algorithms in modern spectral-domain OCT (SD-OCT), it has now been shown that localized (glaucomatous) defects of the retinal nerve fiber layer (RNFL) can be recognized reliably using SD-OCT even before visual field defects become apparent in perimetry.^4^^,^^5^ Today, examining the macula and the peripapillary RNFL using OCT has found its way into routine clinical evaluation for glaucoma. Given the drawbacks of perimetry and the remarkably high resolution of modern SD-OCT, it is reasonable to investigate whether objective measures recorded with SD-OCT can help in the clinical setting to economize the number of visual field tests or to help objectify visual field tests.

Significant research has enhanced our comprehension of the link between structure and function in glaucoma. Statistical or machine learning methods have typically been used to quantitatively assess the association between visual field (VF) measurements and SD-OCT-derived structural measurements, resulting in varying levels of correlation depending on the methodology, model assumptions, and available data. Some studies have demonstrated a moderately high correlation, whereas others have found no association.^6^^–^^8^ Deep learning (DL) techniques have emerged as a promising approach to deepen our understanding of the structure-function relationship in glaucoma, mainly because of their recent success in detecting and predicting ophthalmic diseases.^9^^,^^10^ Current DL-based methods use SD-OCT images as inputs to estimate VF results and have shown promising functional estimates. Christopher et al.^11^ used DL models based on RNFL en-face images, achieving an *R*^2^ of 0.70 and mean absolute error (MAE) of 2.5 decibels (dB) in predicting MD for 24-2 VF tests. They also used DL models from thickness maps from macular OCTs to predict MD for 24-2 VF tests, achieving an *R*^2^ of 0.79 and an MAE of 2.1 dB,^12^ as well as from OCT optic nerve head (ONH) en-face images and RNFL thickness maps, obtaining an *R*^2^ of 0.70 and MAE of 2.5 dB.^11^ Park et al.^13^ developed a DL architecture using a combination of macular and ONH OCTs to obtain a root mean squared error of 4.70 ± 2.56 dB on MD prediction for a cohort of 290 eyes. Yu et al.^14^ performed a longitudinal study on 1678 participants, showing a beneficial contribution to MD prediction (reduction of 0.06 dB of median absolute error) when combining macular and ONH scans with respect to macular scans only. As far as we know, no existing literature currently explores the structure-function relationship resulting from a combination of ONH circle and macular scans on a large patient cohort and at a cluster MD level. Accordingly, our research objective was to investigate whether deep learning algorithms could be used to accurately predict the visual field performance of glaucoma patients by leveraging a combination of SD-OCT images obtained from both the macula and the optic nerve head regions.

The contribution of this work is twofold. First, the VF MD prediction is improved thanks to the combination of macular and ONH OCTs. Second, MD evaluation is refined and extended to specific visual field clusters.

## Methods

### Cohort Description

**Figure 1.**
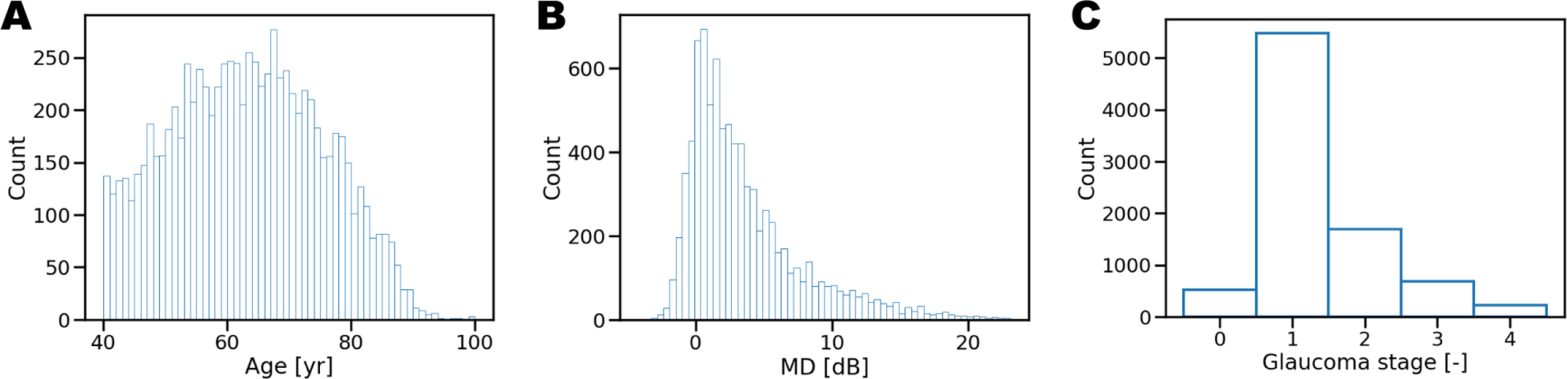
Cohort distributions of age, MD, and GS. (**A**) Cohort age distribution. (**B**) Cohort MD distribution. (**C**) Cohort GS distribution.

In the University Eye Hospital Bern's databank, 8598 OCT-VF triplets from 5238 eyes of 3119 patients were located. The mean patient age was 62.7 ± 12.2 years, and the mean MD of SAP was −3.6 ± 4.3 dB. The distribution between the left and right eyes is symmetrical, with each comprising 50.0% of the total. The distribution of age, MD, and glaucoma stage (GS)^15^ in the tested cohort of eyes are shown in Figure 1 and Table 1.

**Table 1.** Cohort Statistics


Triplets OCT-VF [-]	8598
GS 0 Triplets	526 (6.1%)
GS 1 Triplets	5478 (63.7%)
GS 2 Triplets	1691 (19.7%)
GS 3 Triplets	684 (8.0%)
GS 4 Triplets	219 (2.5%)
Eyes	5238
Patients	3119
Age (yrs)	62.7 ± 12.2
Laterality	50.0%

All examinations and tests were performed at the University Eye Hospital Bern, Inselspital, between 2010 and 2020. The results of a triplet of diagnostic tests, all taken on the same day, had to be available for analysis. This included standard automated perimetry (SAP) performed using an Octopus 900 (Haag-Streit, Köniz, Switzerland) and SD-OCT of the macula and optic nerve head region (Spectralis OCT, Heidelberg Engineering, Germany). Excluded were those eyes that suffered from an already-far-progressed glaucoma. This included eyes with a global peripapillary RNFL < 50 µm because SD-OCT is unreliable to indicate further changes below this critical border.^16^ Eyes with a visual field mean defect above 23.1 dB or a best-corrected visual acuity worse than 0.2 were not included in the performed analysis because these are critical values from a clinical perspective to perform visual field tests reliably. Last, patients not meeting SAP reliability scores (i.e., >20% false-negatives or false-positives during SAP catch trials) or being under 40 years of age were also excluded.

It is worth mentioning that typically VF testing is not conducted in the presence of other ocular comorbidities. However, because of the size of the cohort, conducting a dedicated check on all included samples was not feasible. Given these points, the probability of occurrence of comorbidities in the cohort is considered to be very low.

This study was approved by the University of Bern ethics committee (KEK no.: 2022-01137) and followed the tenets of the Declaration of Helsinki. Informed consent was waived because of the retrospective design of the study.

### OCT Imaging

OCT recordings were performed using a Spectralis OCT. The machine uses a confocal scanning laser ophthalmoscope, obtaining approximately 40,000 A-Scans/minute. The noise was reduced in the recording by activating eye-tracking and setting the repetition rate of each line scan to 26 repetitions. Customary built-in recording algorithms of the OCT machine automatically identified the optic nerve head and macular regions of the eye fundus. This was then used to record OCT line scans parallel to a line connecting the center of the optic nerve head and the center of the macula/fovea regions. The macula was then scanned using 49 or 61 parallel horizontal B-scans capturing the central 30 × 30°. The peripapillary RNFL was scanned using a circular scan centered on the optic nerve head center with a diameter of 3.5 mm.

Three different formats of SD-OCT recordings were used in the study. The first was the 61-slice SD-OCT (61 × 496 × 768 pixels) covering an area of 7.8 × 1.9 × 9.2 mm, covering the complete Early Treatment Diabetic Retinopathy Study (ETDRS) grid. The second recording format was a 49-slice SD-OCT (49 × 496 × 512 pixels) covering an area of approximately 6.6 × 1.9 × 6.5 mm, fully covering the centered ETDRS grid. The third was a 49-slice SD-OCT (49 × 496 × 512 pixels) covering an area of 5.9 × 1.9 × 5.8 mm of the retina. The last scan format was only taken for further analysis in the absence of better-fitting scans covering the ETDRS grid.

**Figure 2.**
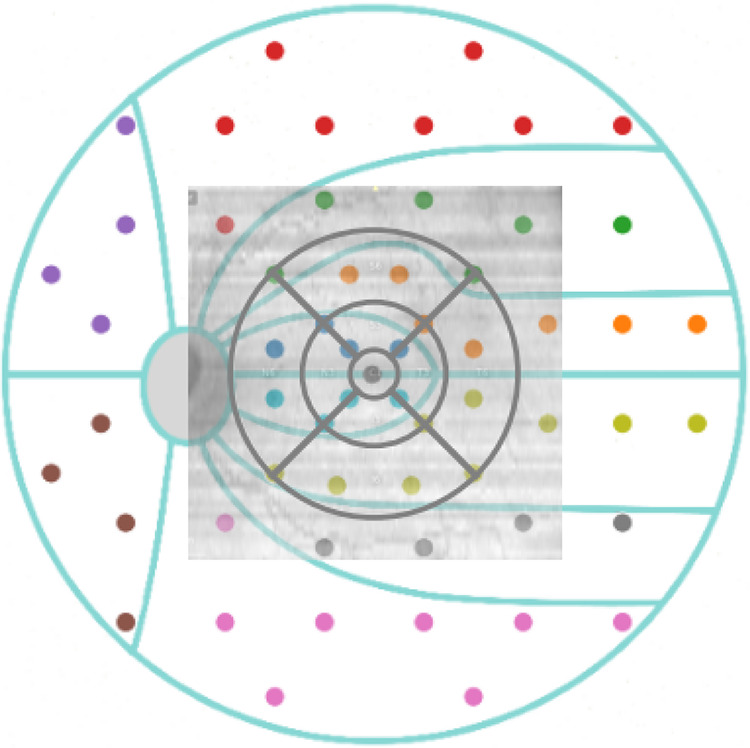
Clustered G pattern test points overlaid with ETDRS grid.

SAP uses the G pattern^17^ to test the central 30° of the visual field. OCT scans the central macular region, which only covers parts of the area tested by SAP (as demonstrated by the square image overlaid in Fig. 2). The ETDRS grid only covers a region of the area scanned by SD-OCT, extended for approximately 10° from the fovea (depicted by the black circle in Fig. 2). Altogether, the ETDRS grid only covers an area of the tested visual field in which a total number of 13 test locations of SAP fall, as shown in Figure 2.

### Mean Defect and Cluster MD Prognosis Models

**Figure 3.**
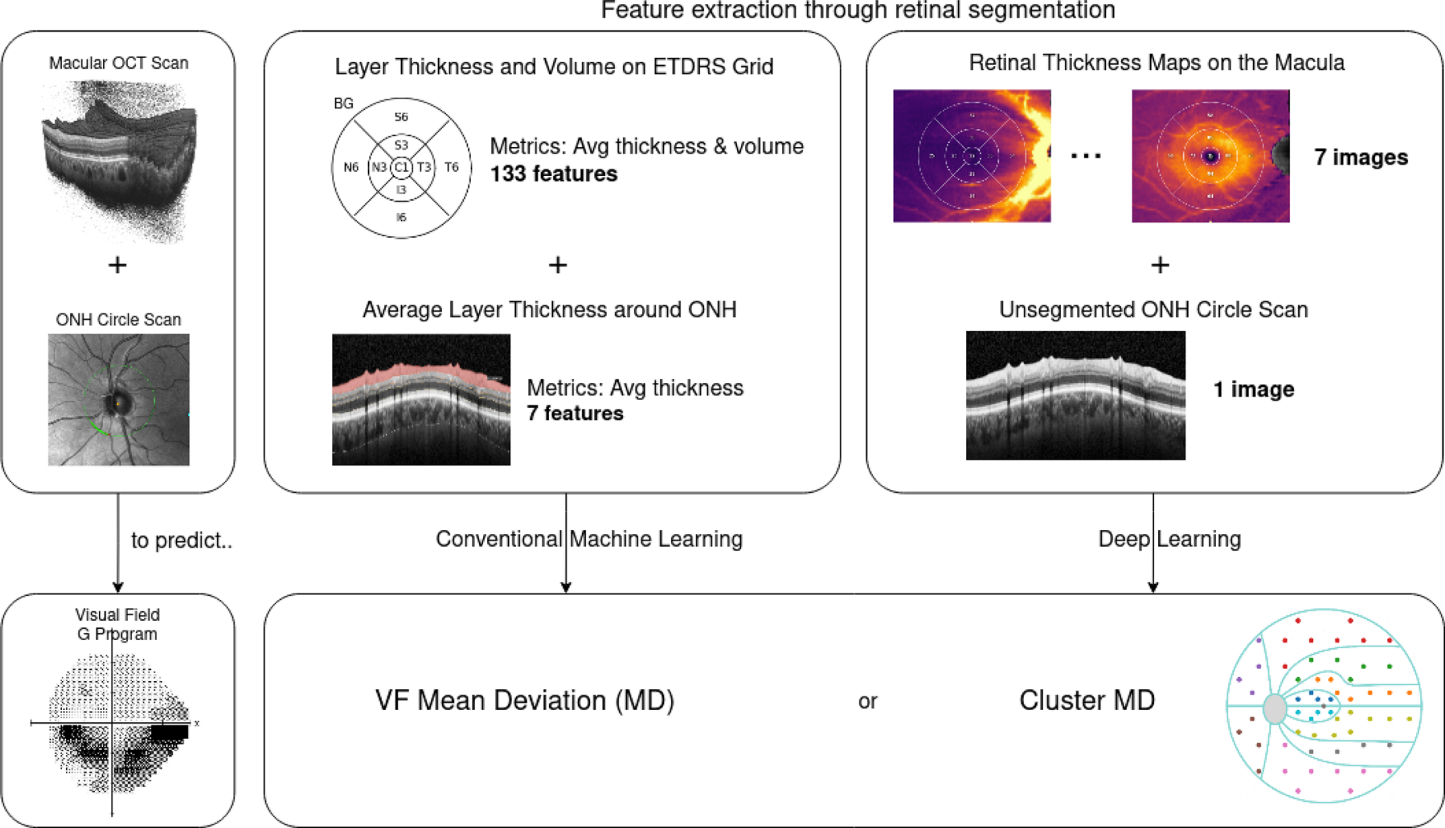
Study workflow for baseline and DL models.

We investigated two problems with significant clinical impacts: the estimation of the MD and the estimation of cluster MDs. Clusters are defined based on the RNFL bundles' entry into the optic disc.^17^ Clusters are numbered from 1 to 10 in a counterclockwise direction starting from the temporal location. Each cluster is represented by a differently colored dot in Figure 2. We evaluated a deep-learning approach described below for both problems and compared it to a baseline model composed of a linear regression model fitted on the retinal layer thicknesses, averaged over the entire ETDRS grid in the case of macular scans. Figure 3 illustrates our processing pipeline.

Macular SD-OCTs were pre-processed using a retinal segmentation algorithm^18^^,^^19^ to extract RNFL, ganglion cell layer and inner plexiform layer (GCL + IPL), inner nuclear layer and outer plexiform layer (INL + OPL), outer nuclear layer (ONL), photoreceptors and retinal pigmented epithelium (PR + RPE), choriocapillaris and choroidal stroma (CC + CS) and total retinal thickness (RT) segmentations. RT is equivalent to the sum of all retinal layers and includes the presence of intraretinal or subretinal fluid and pigment epithelial detachment (PED), if any. Retinal layer segmentations on the macula were exported as thickness map images. The peri-papillary image obtained from the ONH circle scan is used unsegmented. Both image modalities are resized to a 224 × 224 pixel image.

**Figure 4.**
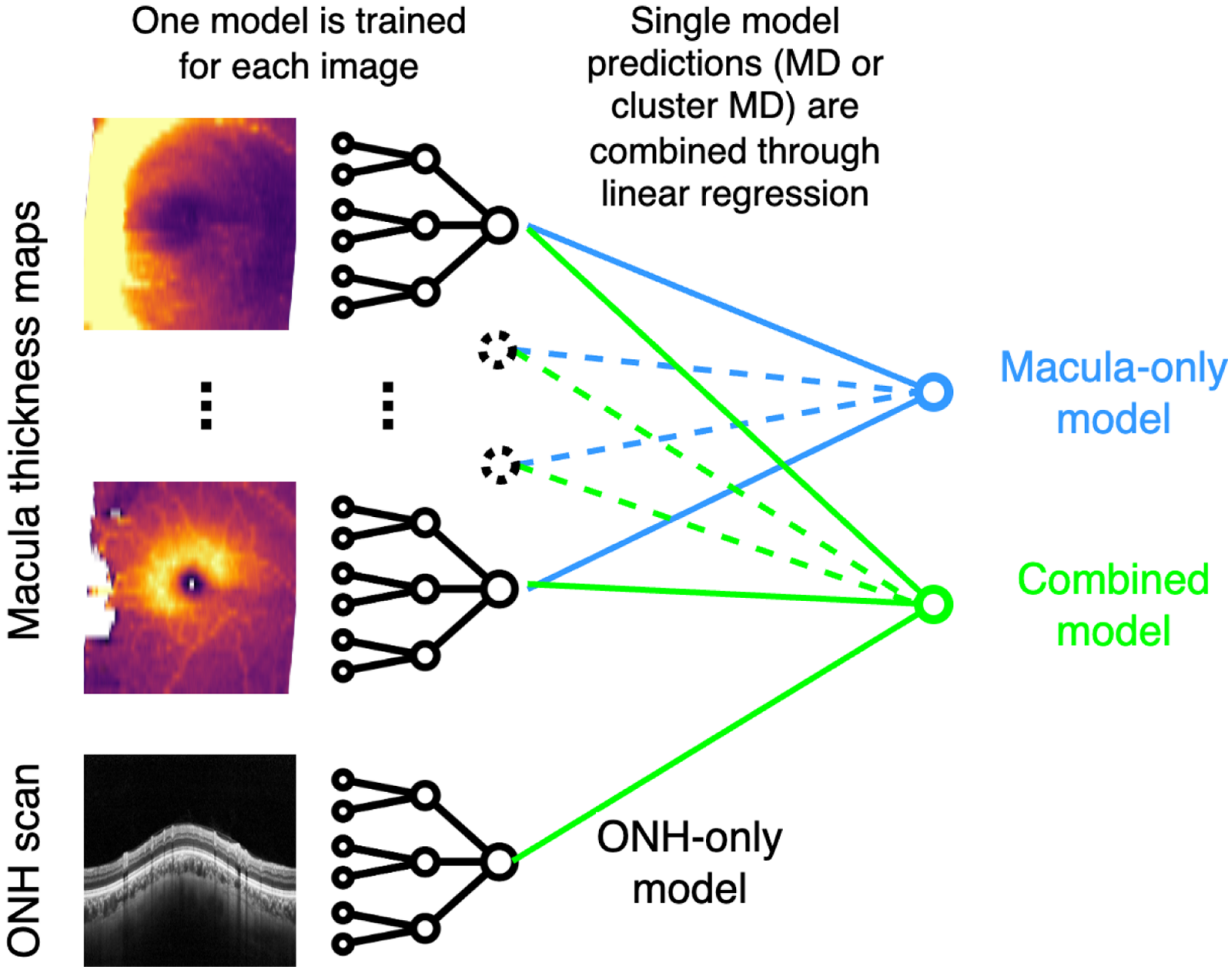
Training strategy for macular-only, ONH-only, and combined models.

We then train one DL model for each retinal layer thickness map and one model for the unsegmented ONH scan. All model predictions are then combined through linear regression to form a unique vectorial or scalar value depending on whether the prediction is made for MD or Cluster MD, as shown in Figure 4. The macular-only model combines all models trained on macular thickness maps. The combined model merges predictions from all macular thickness maps and the ONH scan. Data augmentation is applied as random small rotations, and dropout is adopted to avoid overfitting between training and validation data sets. We use the first fold of the cross-validation set to perform training and validation. We evaluate three different network architectures: ResNet18, ResNet34, and ResNet50, all pre-trained on ImageNet^20^ and either partially frozen—up to layer 3, included—or unfrozen. Models are trained for 50 epochs, tuning the batch size, the learning rate, and the Adam optimizer.

### Evaluation of the DL Models

To test the adopted models, 10% of the obtained groups of datasets were used. The remaining 90% of the datasets were then used for training. The data were stratified by the computed GS to ensure a similar distribution of MD across the training and test data sets. A nonoverlapping condition was enforced at the subject level to avoid the presence of the same subject in both splits. Detailed information is reported in Table 2. Two metrics were used to offer complementary performance insights: *R*^2^ value, which provides a measure unaffected by value magnitude, and MAE, which estimates typical error size.

**Table 2.** Cohort Information on the Used Data Sets, Split by Training and Test

	Training Set	Test Set	Full Data Set
Triplets	7738	860	8598
Eyes	4711	527	5238
Patients	2806	313	3119
Age (yr)	62.6 ± 12.2	62.9 ± 12.1	62.7 ± 12.2
Laterality	50.0%	50.0%	50.0%
Glaucoma stage	1.37 ± 0.82	1.37 ± 0.82	1.37 ± 0.82
MD (dB)	−3.62 ± 4.28	−3.66 ± 4.30	−3.62 ± 4.28
Cluster 1 MD (dB)	−2.74 ± 4.82	−2.84 ± 5.11	−2.75 ± 4.85
Cluster 2 MD (dB)	−3.55 ± 5.50	−3.45 ± 5.31	−3.54 ± 5.48
Cluster 3 MD (dB)	−4.05 ± 5.49	−3.87 ± 5.42	−4.03 ± 5.48
Cluster 4 MD (dB)	−4.95 ± 5.86	−4.92 ± 6.09	−4.94 ± 5.88
Cluster 5 MD (dB)	−3.57 ± 5.37	−3.44 ± 5.54	−3.55 ± 5.38
Cluster 6 MD (dB)	−3.00 ± 5.03	−3.15 ± 5.47	−3.01 ± 5.07
Cluster 7 MD (dB)	−3.65 ± 5.10	−3.79 ± 5.18	−3.67 ± 5.11
Cluster 8 MD (dB)	−3.68 ± 5.31	−3.75 ± 5.25	−3.69 ± 5.31
Cluster 9 MD (dB)	−3.40 ± 5.11	−3.32 ± 5.07	−3.33 ± 5.10
Cluster 10 MD (dB)	−2.74 ± 4.53	−3.16 ± 5.16	−2.78 ± 4.60

We use the trained DL models to generate class activation maps (CAMs)^21^ to highlight areas on the thickness maps and ONH B-scans that most impact the model's decisions. To perform this analysis, we adjusted CAMs to accommodate regression issues by employing an expanded version.^22^ We measure the contribution of each pixel in the input images, and the resulting heatmap is superimposed on the macular thickness maps and ONH B-scans to show which areas of the retina are used model most for predictions. CAMs were only calculated for the test dataset. Images were manually selected to identify cases where the model exhibited accurate or inaccurate predictions (see Figs. 10^11^–12).

**Figure 5.**
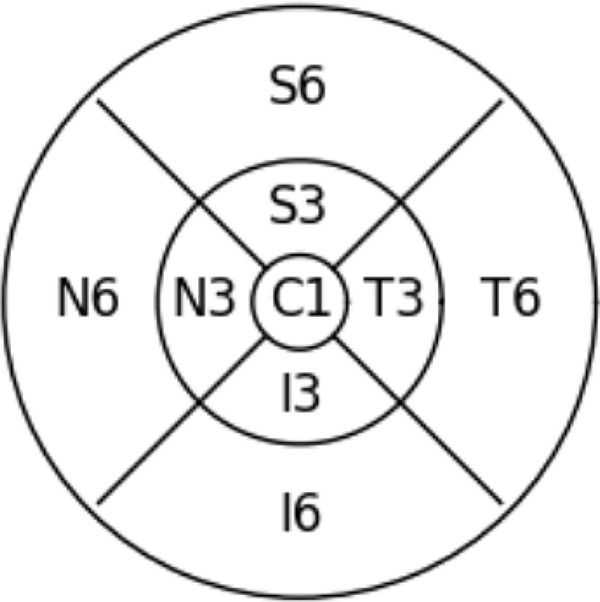
ETDRS grid fields used as inputs for the baseline model.

The proposed model is compared to a baseline model composed of a linear regression model fitted on the layer thicknesses, averaged over the entire ETDRS grid in the case of macular scans. The ETDRS grid is composed of nine subfields, including a central field (C) and eight other areas arranged around and named after their distance and position with respect to the central field (temporal inner and outer: T3, T6; nasal inner and outer: I3, I6; superior inner and outer: S3, S6; inferior inner and outer: I3, I6), as depicted in Figure 5.

ONH scans were pre-processed using the same segmentation algorithm and averaged over the peri-papillary region to obtain average thickness values for each considered retinal layer. In total, we thus compute 7 (retinal layers on the macula) × 9 (ETDRS regions) + 7 (retinal layers on the ONH) = 70 segmentation-based morphological features per triplet. The training procedure for those models is based on a grid search evaluated on a fivefold cross-validation.

## Results

### MD Prediction

**Figure 6.**
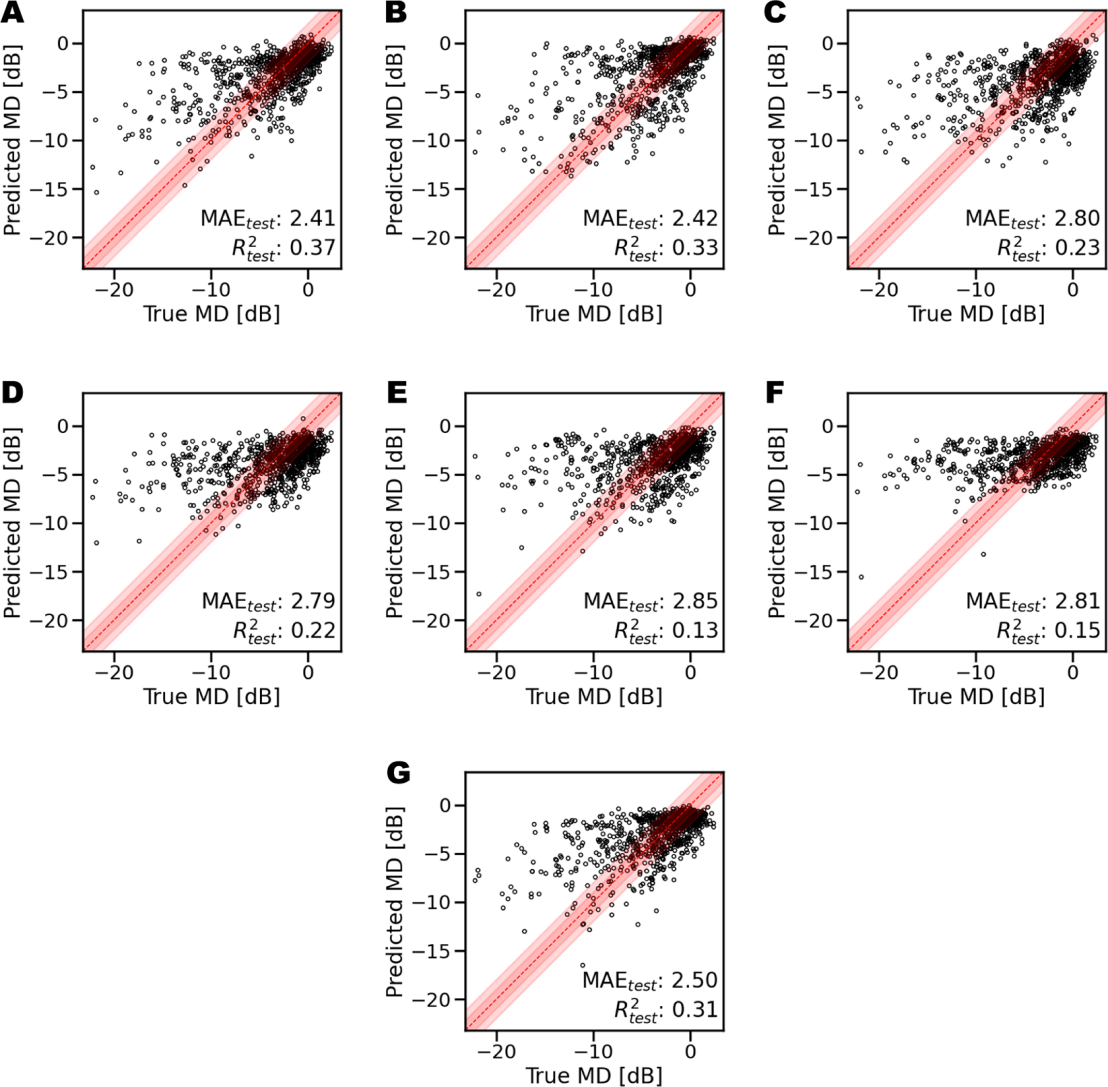
Scatterplots showing the correlation between measured values and deep learning G-pattern visual field (VF) mean deviation (MD) estimates. Resnet18 trained on specific retinal macular thickness maps. The red band highlights the ±2 dB region. (**A**) retinal nerve fiber layer (RNFL). (**B**) ganglion cell layer and inner plexiform layer (GCL + IPL). (**C**) inner nuclear layer and outer plexiform layer (INL + OPL). (**D**) Outer nuclear layer (ONL). (**E**) photoreceptors and retinal pigmented epithelium (PR + RPE). (**F**) choriocapillaris and choroidal stroma (CC + CS). (**G**) retinal thickness (RT).

**Figure 7.**
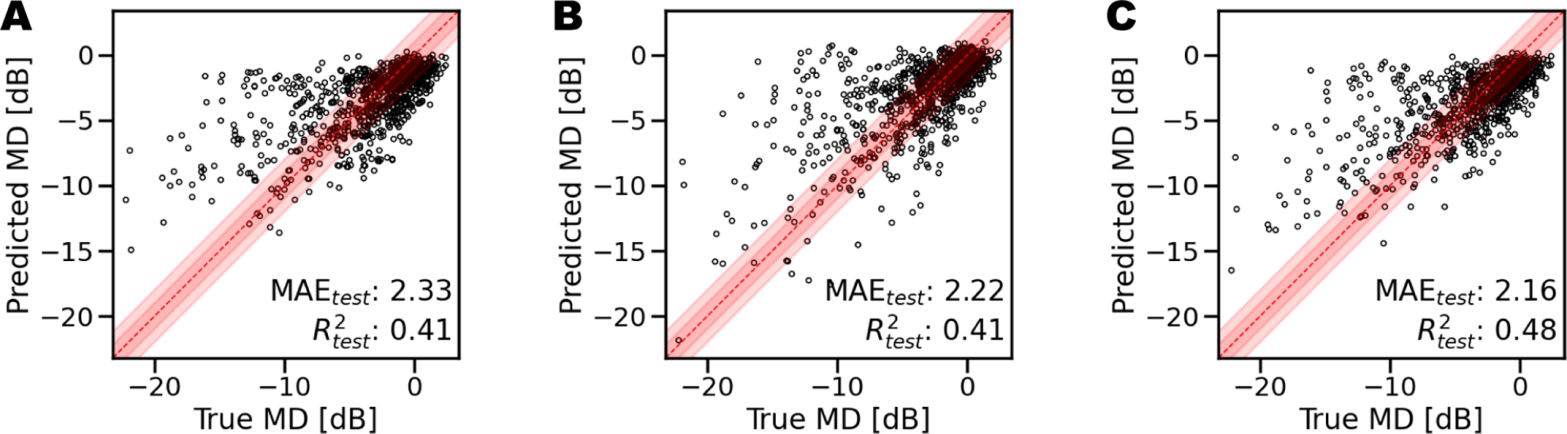
Scatterplots showing the correlation between measured values in decibels and deep learning estimates of G-pattern VF mean deviation (MD). (**A**) Macula-based predictions. (**B**) ONH-based predictions. (**C**) Combined predictions.

Among the deep learning models considered for this study, the ones providing the overall best performance are Resnet18 architectures for macular thickness maps and Resnet50 for ONH OCT scans. The best performance was obtained with models pre-trained on ImageNet and freezing the first three layers to control overfitting, with a batch size of 8 and a learning rate of 10^−^^4^. Figure 6 depicts the truth-prediction plots for all the models trained on individual thickness maps. The retinal layers achieving the best performance in MD regression are RNFL, GCL + IPL, and RT, with a regression score of 0.37, 0.33, and 0.31 and a MAE of 2.41, 2.42, and 2.50, respectively. Figure 7 shows the ground truth-prediction plots obtained considering macular thickness maps, peri-papillary scans, or a combination of the two. The best predictions based on macular thickness obtained *R*^2^ = 0.41 (MAE = 2.33 dB), on peri-papillary scans *R*^2^ = 0.41 (MAE = 2.22 dB) and combined macular and peri-papillary scans *R*^2^ = 0.48 (MAE = 2.16 dB).

**Figure 8.**
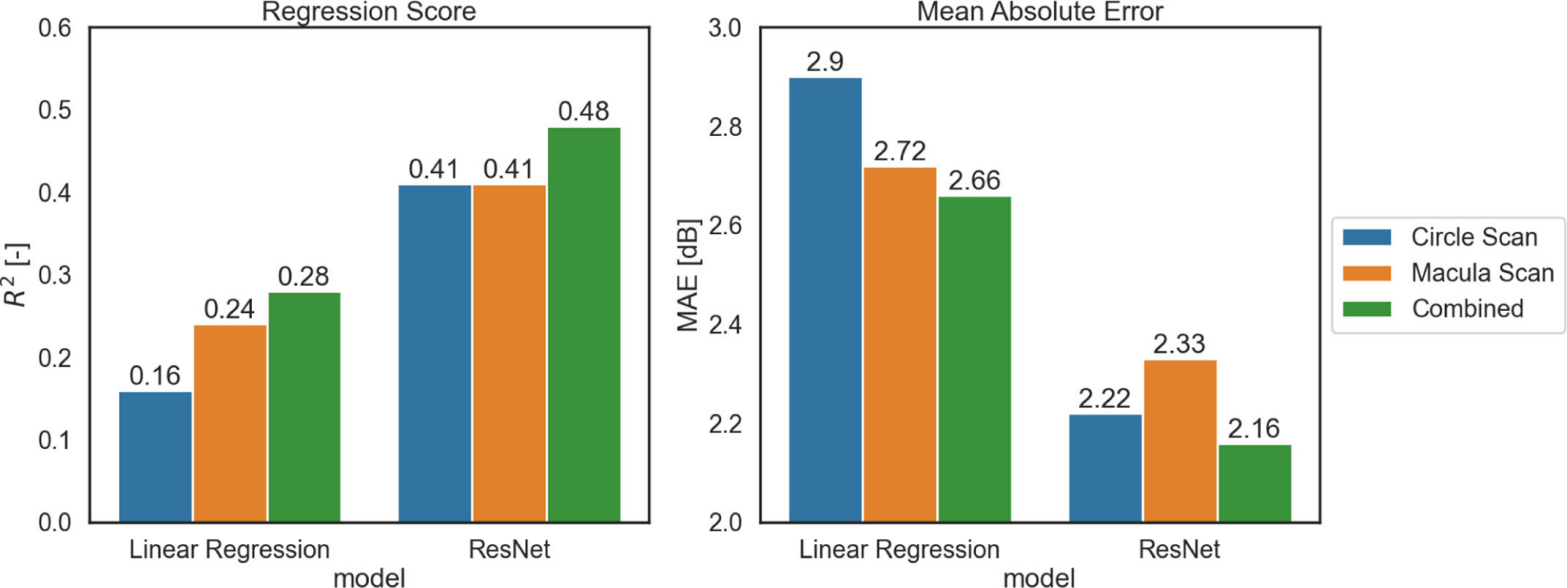
Summary MAE and *R*^2^ for the baseline linear regression model and ResNet on MD prognosis. ResNet18 was used for macula scans, and ResNet50 was used for ONH circle scans. Combined models were obtained by linear combination of single predictions.

Figure 8 compares the best results obtained with Resnet with a baseline regression model. Our proposed method outperformed the baseline model for macular scans (*R*^2^ = 0.41 vs. *R*^2^ = 0.24, MAE = 2.33 dB vs. MAE = 2.72 dB), peri-papillary scans (*R*^2^ = 0.41 vs. *R*^2^ = 0.16, MAE = 2.22 dB vs. MAE = 2.90 dB), and combined scans (*R*^2^ = 0.48 vs. *R*^2^ = 0.28, MAE = 2.16 dB vs. MAE = 2.66 dB).

**Figure 9.**
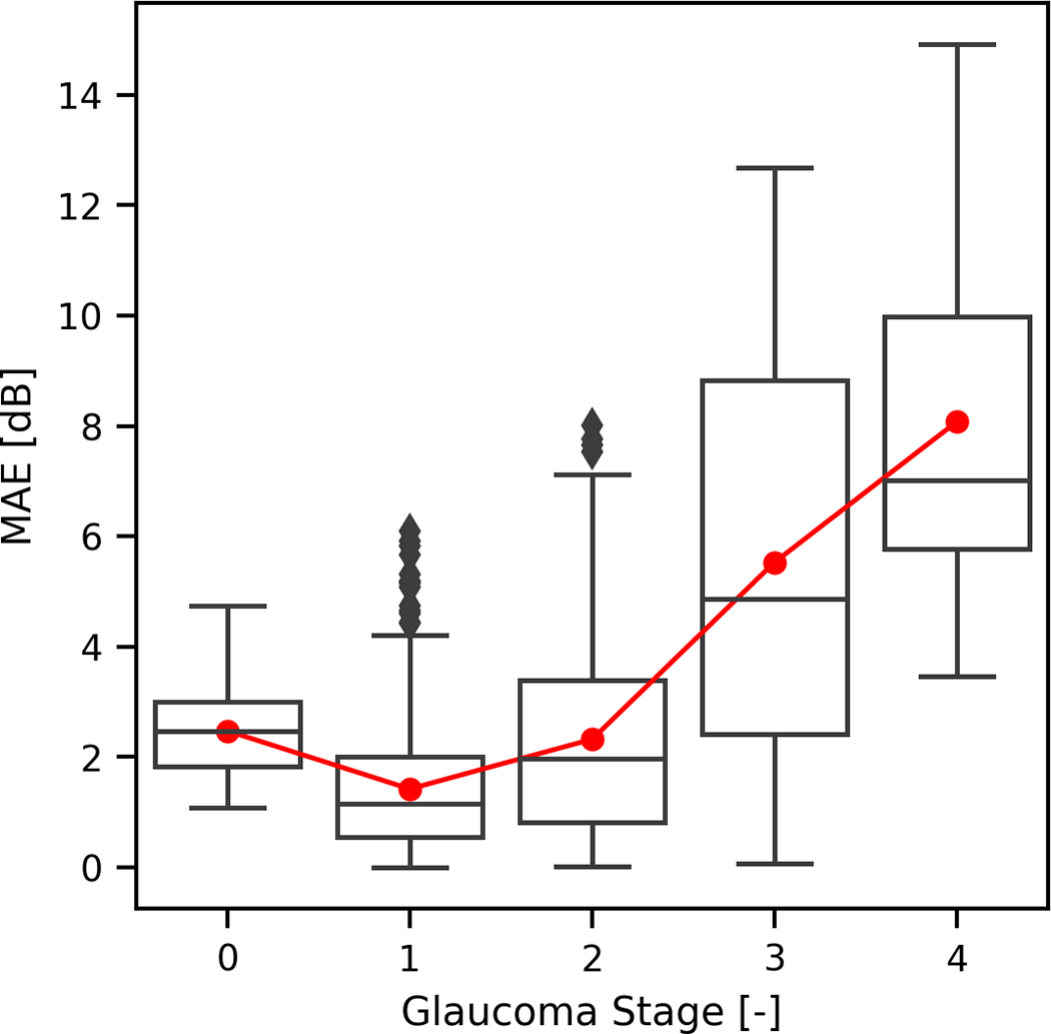
Boxplots related to sample-wise absolute error of our best model across different GS values. Red line depicts MAE performance across the GS spectrum. Metrics computed on the test set.

When assessing the combined model across various glaucoma stages, it demonstrates its strongest performance in predicting early and moderate glaucoma (GS 1 and 2). However, its performance diminishes for both GS 0 and severe glaucoma cases (GS > 2). Additional information and insights on this point can be found in Figure 9 and Table 3.

**Table 3.** MAE Values Across Different GS Values

GS	0	1	2	3	4
MAE (dB)	2.47	1.42	2.32	5.53	8.08

Metrics computed with our best model on the test set.

### Contributors to the Final Prediction

**Figure 10.**
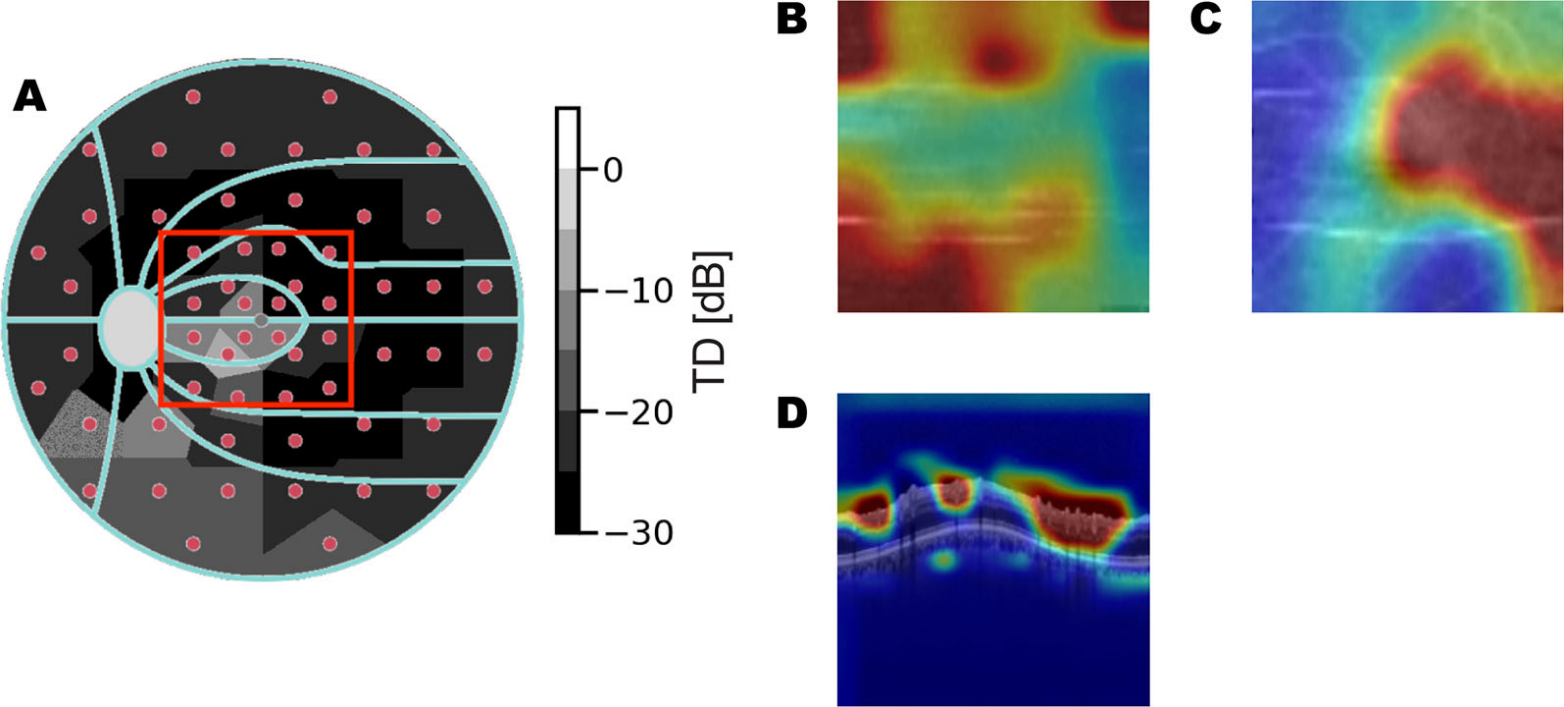
Eye with severe glaucoma. Actual MD: −22.20 dB. Predicted MD: −16.18 dB. (**A**) Measured total deviation [dB] from VF examination. The red box shows an overlapped OCT scan region. (**B**) CAM of RNFL thickness map. (**C**) CAM of GCL + IPL thickness map. (**D**) CAM of ONH circle scan.

To better understand how different features contribute to the final MD estimation, informative image regions are shown by overlaying CAMs on the input image (see Figs. 10^11^–12). The results show retinal layers with the highest *R*^2^ scores, along with ONH B-scans. Images of retinal thickness maps, unsegmented ONH scans, and CAMs that correspond to MD estimations of G-pattern VF are presented as examples, including both GS 0 and glaucomatous eyes.

**Figure 11.**
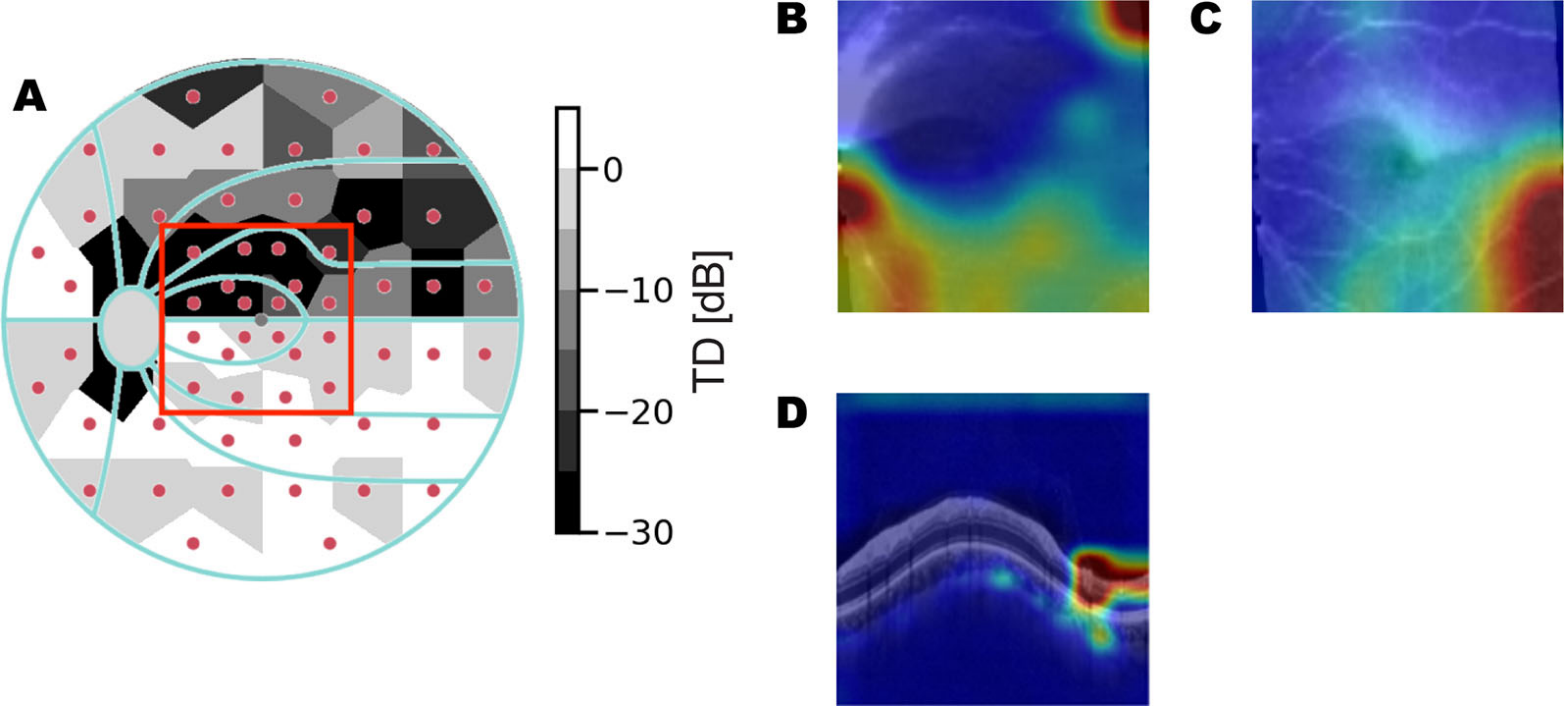
Eye with mid glaucoma. Actual MD: −8.00 dB. Predicted MD: −6.98 dB. (**A**) Measured total deviation [dB] from VF examination. Red box shows overlapped OCT scan region. (**B**) CAM of RNFL thickness map. (**C**) CAM of GCL + IPL thickness map. (**D**) CAM of ONH circle scan. Activation present in temporal region.

**Figure 12.**
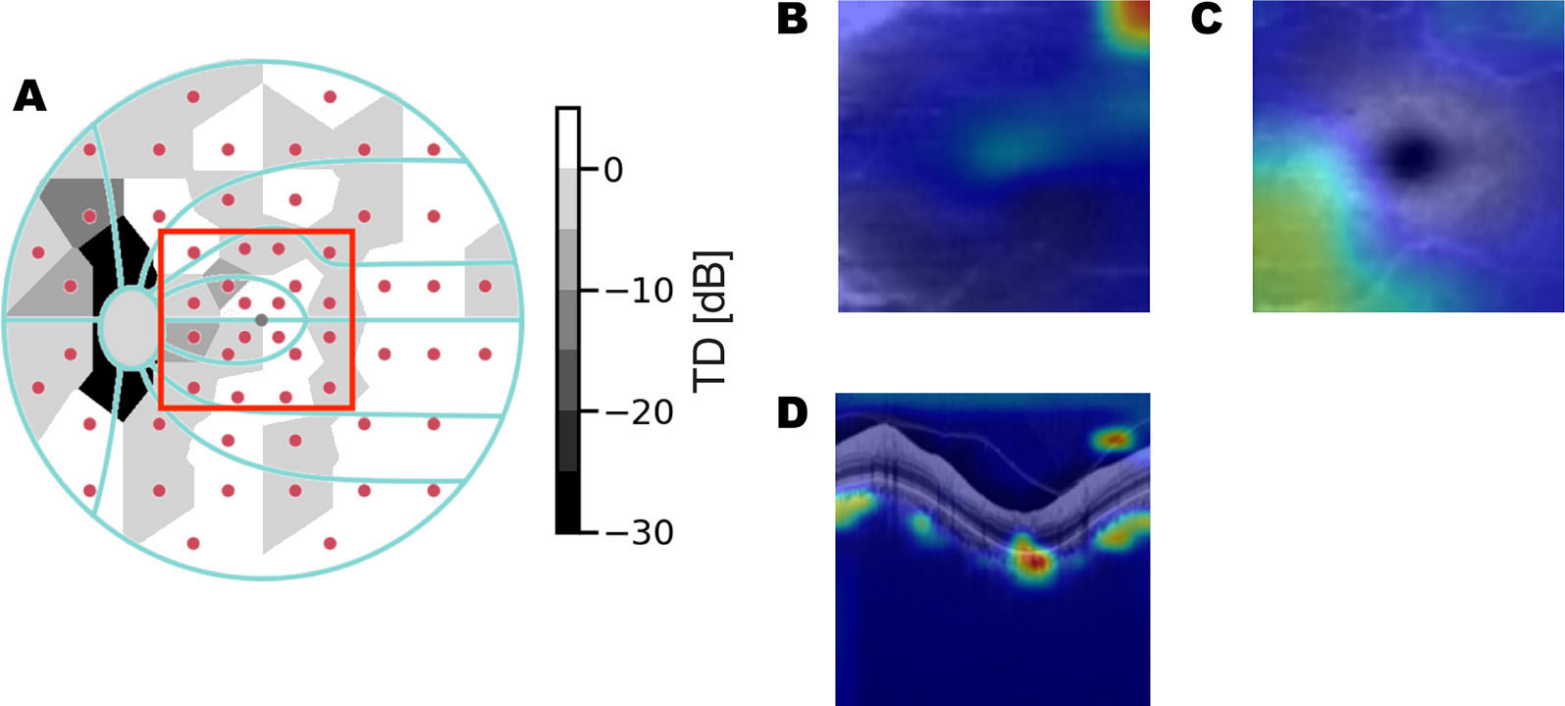
Eye with early glaucoma. Actual MD: −0.60 dB. Predicted MD: −1.80 dB. (**A**) Measured total deviation [dB] from VF examination. Red box shows overlapped OCT scan region. (**B**) CAM of RNFL thickness map. (**C**) CAM of GCL + IPL thickness map. (**D**) CAM of ONH circle scan.

### Predicting Cluster MD

The computation of MD was extended to different region clusters related to glaucoma to identify regions of variability in model performance across the visual field. Table 4 and Table 5 list the regression score and MAE for baseline and deep learning models trained with information from the macula and ONH scans. This result is also visualized in Figure 14.

**Table 4.** *R* ^2^ Score of Cluster MD Prediction for Baseline and Deep Learning Models

Cluster	1	2	3	4	5	6	7	8	9	10
Baseline	0.27	0.30	0.24	0.18	0.09	0.12	0.18	0.25	0.34	0.25
Deep Learning	0.44	0.50	0.47	0.28	0.21	0.27	0.36	0.46	0.56	0.41

**Table 5.** MAE (dB) of Cluster MD Prediction for Baseline and Deep Learning Models

Cluster	1	2	3	4	5	6	7	8	9	10
Baseline	2.77	3.00	3.35	4.24	3.86	3.40	3.28	3.19	2.80	2.76
Deep Learning	2.36	2.33	2.69	3.94	3.38	3.00	2.90	2.64	2.12	2.34

Figure 15 depicts the single contributions of macular and optic disc information to the overall regression performance of the combined model. ONH circle scans contribute to MD predictions in the peripheral visual field regions, specifically in the inferior and nasal regions, where the increase in regression score obtained by integrating ONH-based predictions in the model is quantified in 0.13 for cluster 6. For the prediction of MD in clusters 1, 2, 9 and 10, the combined model relies solely on the information related to the retinal layers on the macula. When estimating cluster MD, our combined DL approach showed the best results in predicting central clusters, achieving an *R*^2^ value of 0.56 (MAE, 2.1 dB) for MD on cluster 9, located in the central-temporal visual field region.

## Discussion

Accurate estimation of visual function from structure can improve individualized glaucoma care by allowing clinicians to adjust the frequency of VF testing using DL estimates of functional damage. VF testing can be expensive, time-consuming, and produce variable results, leading to multiple tests and delayed diagnosis of glaucoma. Accurate estimation of visual function from SD-OCT imaging can identify disease earlier and determine progression faster, enabling individualized VF testing frequency and reducing the overall need for VF tests. DL models can estimate changes in VF results and postpone or recommend further testing, providing cost savings and standard metrics for monitoring patient visual function while reducing reliance on VF testing.

The proposed DL method accurately estimated G-pattern VF MD using macula-centered SD-OCT scans and ONH circular scans, outperforming estimates based on linear regression mean layer thicknesses. Specifically, in assessing G-pattern VFs, our combined DL approach showed an R^2^ value of 0.48 (MAE = 2.16 dB) for MD. Individually, the macular RNFL and GCL + IPL layers and the whole macula thickness showed the highest performance in estimating G-pattern MD. PR + RPE and CC + CS were the worst-performing layers. As explained in the methods section, the macula-combined model included a linear regression step that contributed to an increase in performance compared to the single macular retinal layers taken individually. However, the best performance obtained from RNFL (*R*^2^ = 0.37, MAE = 2.41 dB) did not differ substantially from the macula-combined model performance (*R*^2^ = 0.41, MAE = 2.33 dB). This suggests that layers may contribute different information regarding function in glaucoma, among which macular RNFL is the highest. The total retinal thickness was among the three most informative layers in all cases, as depicted in Figure 6. The retina contains information about several additional layers not typically monitored in glaucoma. Combining the macula information with optic nerve head scans proved beneficial (0.07 on *R*^2^, −0.16 dB on MAE).

Figure 8 summarizes the findings described so far:

1. Deep learning outperforms the baseline model built on ETDRS average thickness values. The higher complexity and information in the features mainly explain such results.
2. Higher model complexity and added information contained in macular and peri-papillary images with respect to scalar thickness and volume values are the main factors contributing to a performance increase.
3. Combining ONH and macular information of the eye retinal structure proves beneficial for all models considered in the current study regarding regression score and mean absolute error.

Moreover, in the comprehensive evaluation of the model performance across diverse stages of glaucoma, it shows its best performance in the prediction of early and moderate glaucoma (GS 1 and 2). However, its effectiveness diminishes notably in instances involving both glaucoma suspects and those afflicted with severe glaucoma.

It is plausible that these observations reflect the clinical understanding of the structure-function relationship in glaucoma. It's worth noting that they also could be explained by differences in dataset sizes among glaucoma stages, potentially providing more training data (Table 1) for stages where the model performs better. However, this remains a hypothesis and is not definitively established.

**Figure 13.**
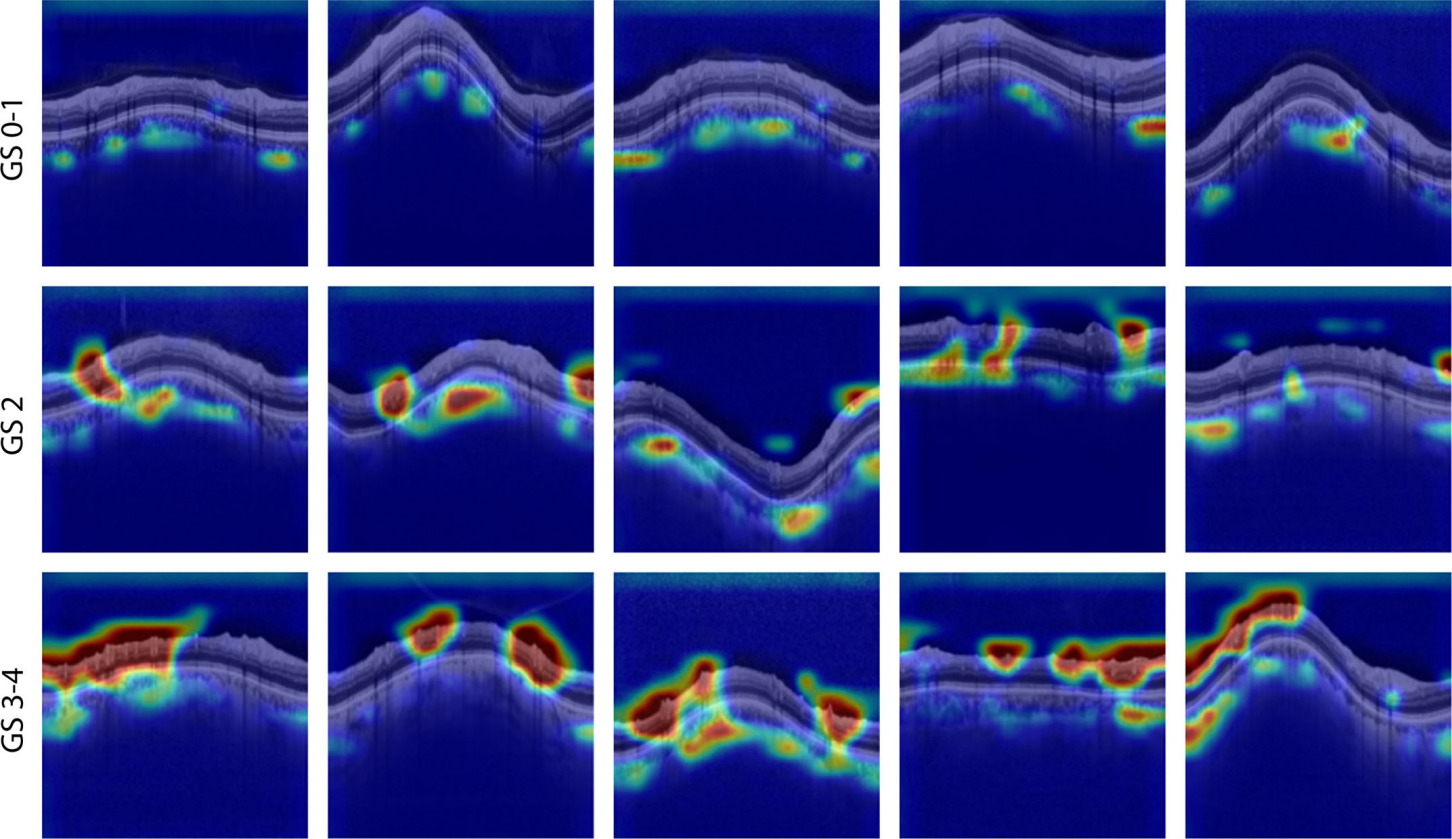
CAMs overlaid on representative peri-papillary scans for different glaucoma stages. The same color scale was used for all CAMs.

We used CAM techniques to locate the regions of thickness maps the DL models used for their estimations, which helped us comprehend their functioning. They provided examples of RNFL and GCL + IPL thickness maps, ONH scans, activation maps, and the corresponding VF outcomes. The CAMs conducted on ONH Bscans indicated that for mid-severe and early-stage glaucoma, the peri-papillary CC + CS and RNFL layers played a crucial role in MD prediction, as shown in the CAMs in Figure 13^14^,^15^. The CC+CS layer is rarely mentioned as a biomarker for VF damage or a layer used in glaucoma management. However, there is evidence in the literature that choroidal structural changes in glaucoma are increased for patients with open-angle, normal-tension, and pre-perimetric glaucoma and are not necessarily associated with central retina dysfunction.^23^^–^^26^

**Figure 14.**
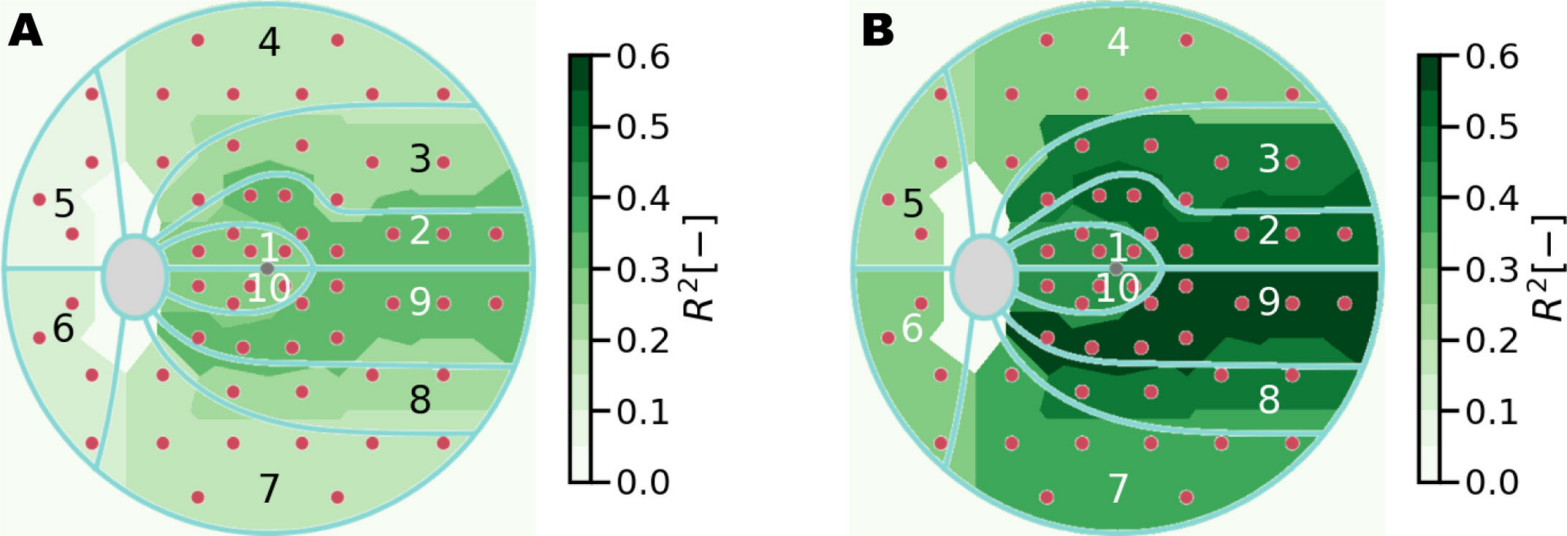
Regression score for cluster MD prediction. Comparison between linear regression baseline model and the proposed method. (**A**) Baseline model. (**B**) Deep learning model.

**Figure 15.**
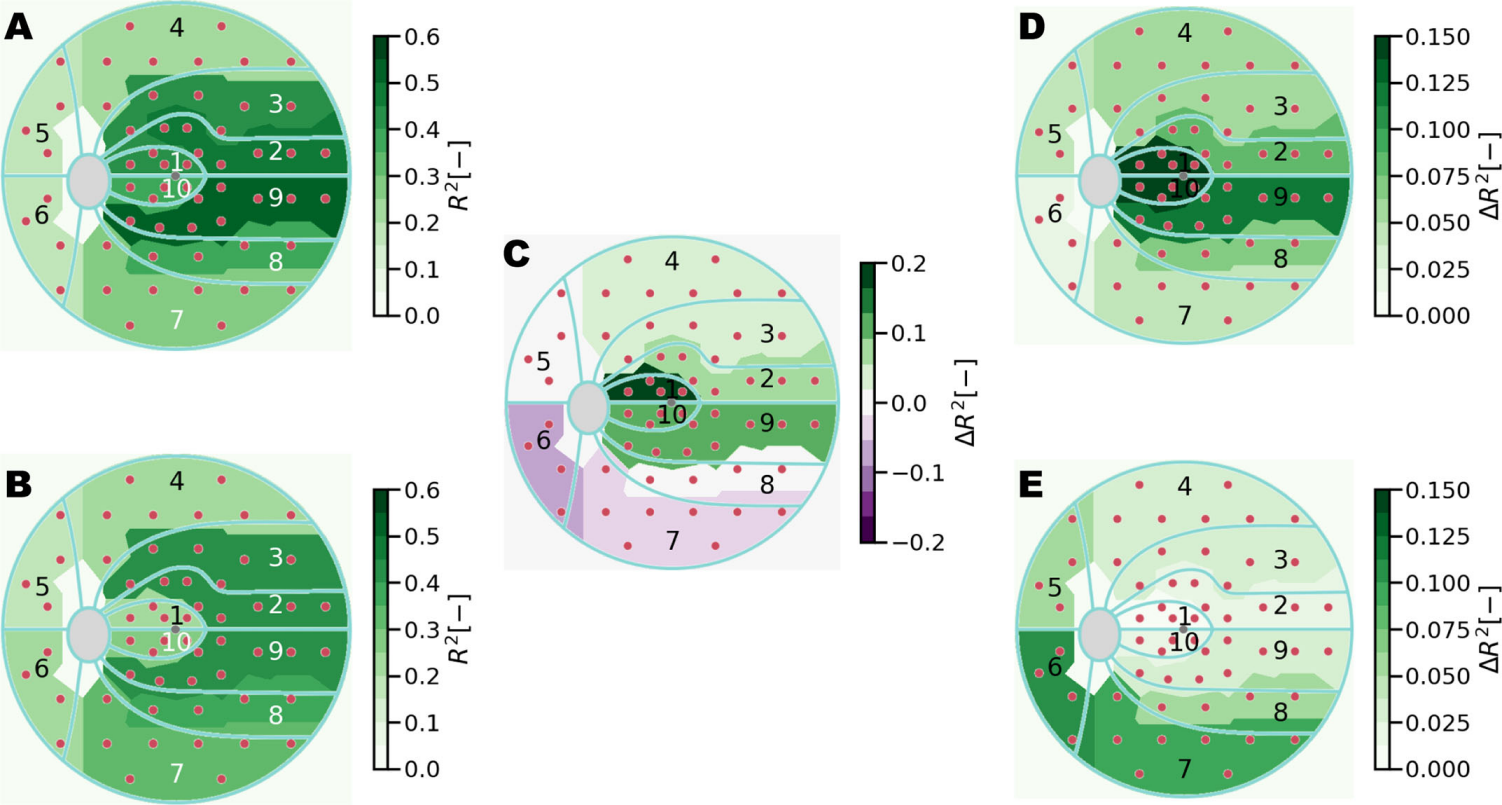
Comparison between regression score on cluster MD achieved by macula and ONH information, relative to combined performance. (**A**) Regression score of macula-based prediction. (**B**) Regression-score of ONH-based prediction. (**C**) Difference in regression score between macula-based and ONH-based prediction. Green areas indicate a higher regression score for macula-based prediction. (**D**) Contribution of macula-based prediction to ONH-based prediction, expressed in terms of regression score. (**E**) Contribution of ONH-based prediction to macula-based prediction, expressed in terms of regression score.

The baseline model, trained using retinal layers' average thickness on the macula and ONH, yields low regression scores and cannot differentiate functional loss among clusters. The reason is likely because of the highly condensed information represented by the input features. On the other hand, DL outperforms the baseline model for all the clusters. However, it performs poorly towards the peripheral clusters, especially in the superior, inferior, and nasal regions. The higher regression score is explained by the spatial information contained in ONH images with respect to average layer thickness values used as input for the baseline model. As expected, the central fields achieve the highest regression score, with the outer clusters (4 to 7) being the worst. Clusters 1 and 10, despite being in the center region of the visual field and fully covered by the macular OCTs, are less predictable than the surrounding clusters.

Several groups have previously applied DL strategies to estimate VF outcomes, achieving results similar to those of the current study, proving that regression performance strongly depends on the MD distribution of the study cohort. Christopher et al.^12^ used thickness maps of retinal layers on the macula to predict 24-2 VF MD. They performed well (*R*^2^ = 0.79) on a cohort with a mean MD = −4.5 dB. They also showed how the prediction performance decays (*R*^2^ = 0.28) in cohorts including early-glaucoma cases (mean MD = 1.3 dB). Considering these results, our method shows good performance (*R*^2^ = 0.48) on a cohort including glaucoma suspects, early-, mid-, and severe-glaucoma stages (mean MD = −3.6 dB) on a broader VF test pattern than the cited studies.

Some limitations to this study need to be considered when considering our results. First, it relied on automatically extracted layer segmentations to generate thickness maps to use as input for the DL models. This means the models can be sensitive to segmentation errors, with erroneous segmentation leading to inaccurate estimates. It should be noted that the quality and segmentation of the images could not be reviewed manually due to the amount of collected data. Another area for improvement is the unknown generalizability of the results presented to other populations. Generalizability of the DL models to external datasets was not possible due to the high specificity of the dataset used for this study. The study population collected may not be representative of other datasets in terms of age, race, collection protocols, or some other unknown confounding variable, and the models may have learned structure-function relationships specific to these data. Macular input modalities present high heterogeneity of the portion of the macula covered by the scans. The variation of the amount of macula captured during acquisition affects both training performance and the degree of overlap with the visual field. Also, the G-pattern for visual field testing is very large and estimating the functional information over such an extended region is challenging. An alternative could be to use a narrower pattern (i.e., 24-2), which may improve the estimation performance because of a higher overlap between structural and functional information.

The rationale behind using thickness maps for macular OCTs and unsegmented images for ONH OCTs lies in the intricacy and multidimensionality of unsegmented macular volumes. These volumes contain a vast amount of data, necessitating complex models to identify significant patterns effectively—a level of complexity beyond the scope of the current study. Thus, to streamline the data and facilitate model interpretation, the decision was made to reduce the information in macular OCT volumes through segmentation preprocessing, ultimately utilizing thickness maps as model inputs. It is worth noting that this approach was not required for ONH scans because they are acquired in single-B-scan mode. Additionally, applying a preprocessing step to ONH scans would yield a thickness vector for each considered layer in the study. Hence, unsegmented image data sufficed for ONH scans, eliminating the need for segmentation preprocessing. Our future work will investigate using unsegmented macular scans to reduce the reliance on segmentation as a preprocessing step.

This study proposed a DL algorithm to investigate the structure-function relationship in glaucoma patients. We showed that using our DL approach to extract relevant information from OCT images could lead to new biomarkers for clinical decision-making and improve personalized patient care. DL-based estimates proved to be accurate in determining functional loss based on VF metrics, with model accuracy depending on the severity of functional loss as supported by our results and the existing literature. These techniques offer clinicians a more precise way of estimating function from SD-OCT images and tailoring the frequency of VF testing to each patient's needs. Therefore, given the widespread use of SD-OCT imaging in glaucoma care, it is crucial to take advantage of DL to extract as much relevant information as possible from the images.
